# Health Experiences of Sexual and Gender Minority People Living With Dementia and Their Care Partners: Protocol for a Scoping Review

**DOI:** 10.2196/44918

**Published:** 2023-03-13

**Authors:** Jennifer T May, Melissa Louise Harris

**Affiliations:** 1 School of Nursing Duke University Durham, NC United States; 2 Center of Innovation to Accelerate Discovery and Practice Transformation Durham VA Healthcare System Durham, NC United States

**Keywords:** lesbian, gay, bisexual, transgender, and queer, LGBTQ, dementia, caregiver, identity, gender minority, health inequity, vulnerable population, health care, health disparity, scoping review protocol, sexual minority

## Abstract

**Background:**

People living with dementia and their care partners who identify as a sexual and gender minority (SGM) often experience specific health inequities and disparities due to discrimination related to age, cognitive impairment, and being SGM.

**Objective:**

The purpose of this scoping review is to identify, explore, and synthesize the state of the science regarding the health and health care experiences of SGM people living with dementia and their care partners. This review also aims to identify gaps in research and set forth key recommendations to improve the health and health care experiences of SGM people living with dementia and their care partners by advancing health equity through research, policy, and practice.

**Methods:**

The protocol follows the guidelines set forth by Joanna Briggs Institute protocol for scoping reviews. Steps of this framework that will be followed include (1) identifying the research question; (2) identifying relevant studies; (3) study selection; (4) charting the data; (5) collating, summarizing, and reporting the results; and (6) consultation. This scoping review will explore several electronic databases, including MEDLINE, Embase, CINAHL, AgeLine, PsychINFO, and Scopus. Health librarians will conduct the initial search for articles that are in English, include people living with dementia who identify as SGM, SGM people living with dementia and their care partners, or SGM care partners caring for people with dementia. Studies must be peer reviewed and focus on the phenomenon of interest, which is the health and health care experiences of participants. Covidence will be used to review abstracts and full-text articles and to screen articles. After the search has been completed, 2 independent reviewers will screen article titles and abstracts to identify eligibility. Discrepancies will be discussed and decided upon by the 2 reviewers. Relevant studies will be collected, and data will be extracted and charted to summarize key findings. Key findings will be presented to a community stakeholder group of SGM care partners and people living with dementia, and a listening session will be convened.

**Results:**

This scoping review will identify the state of the science of health and health care experiences of people living with dementia and their care partners who identify as SGM. We will identify gaps and provide recommendations to inform future research, policy, and practice to improve health and health care experiences of SGM people living with dementia and their care partners.

**Conclusions:**

Little is known about people living with dementia and their care partners who identify as SGM. This scoping review will be one of the first to identify the health and health care experiences of people living with dementia and their care partners who identify as SGM. The results of this review will be used to guide future interventions and to inform future policy and practice to improve health care and reduce health disparities in this population.

**International Registered Report Identifier (IRRID):**

PRR1-10.2196/44918

## Introduction

Older adults are often at the intersection of multiple minoritized identities due to stigma surrounding aging and age-related conditions [1]. Older adults who identify as lesbian, gay, bisexual, transgender, queer, as well as other sexual or gender minority (SGM) identities are known to experience unique, compounding stressors and resultant disparities in health outcomes (eg, depression, anxiety, posttraumatic stress disorder, cognitive impairment, cardiovascular disease, hypertension, and diabetes) due to discrimination, stigma, and structural inequities [2-5]. Prior research supports older adults living with dementia and their care partners experience disparities in health outcomes due to individual (eg, grief, comorbid conditions, as well as limitations with physical and cognitive abilities) [6-8] and social stressors (eg, declining social networks and relationship strain) [9-11] that are distinct from the already complex, stigmatized process of aging [12]. People living with dementia and their care partners who identify as SGM represent a unique intersecting population at risk of experiencing health disparities and care inequities due to longstanding false beliefs and discrimination surrounding aging, cognitive impairment, and SGM status.

Many SGM older adults experience health care access barriers, including nonaffirming providers that can delay pursuit of general and specialist care [2]. Care partners of SGM older adults also experience barriers to providing optimal care due to discrimination, providers that may not acknowledge nontraditional partnerships, and health care systems and interventions that do not promote health equity or address social determinants of health [13,14]. Although such barriers are known to affect health outcomes among SGM older adults, little is known about the unique health and health care experiences of SGM people living with dementia and their care partners. This knowledge gap is especially concerning considering that the number of SGM people living with dementia and those providing care to them is anticipated to rise in the coming decades as the population ages [15].

Understanding the health and health care experiences of SGM people living with dementia and their care partners is critical to elucidating health disparities and inform targeted strategies to better support families living with dementia. To date, no prior review has synthesized the evidence surrounding people at the intersection of being SGM and living with dementia or caring for someone with dementia. Foundational knowledge and recommendations to inform policy initiatives that promote health equity for SGM people living with dementia are needed [16,17]. The purpose of this scoping review is to identify, explore, and synthesize the state of the science regarding the health and health care experiences of SGM people living with dementia and their care partners. This review also aims to identify gaps in research and set forth key recommendations to advance health equity by improving the health and health care experiences of SGM people living with dementia and their care partners through research, policy, and practice.

## Methods

### Procedure

A scoping review framework will be used broadly to explore the state of the science pertaining to SGM people living with dementia and their care partners, which represent a historically underexamined group of older adults [18]. The Joanna Briggs Institute protocol for scoping reviews [19], which is informed by the advancing methods of Levac, Colquhoun, and Peters will be used to guide the scoping review process [20,21]. Steps of this framework that will be followed include (1) identifying the research question; (2) identifying relevant studies; (3) study selection; 4) charting the data; (5) collating, summarizing, and reporting the results; and (6) consultation. Consultation is an optional step of the framework that will be addressed in this review. Adherence to the PRISMA-ScR (Preferred Reporting Items for Systematic Reviews and Meta-Analyses extension for Scoping Reviews) guidelines will be used to promote rigor in this review [22].

### Step 1: Identifying the Research Question

This review is guided by the following research questions:

- What is the extent and nature of research relating to the health and health care experiences of SGM people living with dementia and their care partners?
- What gaps exist in the evidence surrounding health and health care experiences of SGM people living with dementia and their care partners?
- What areas in research, policy, and practice warrant further exploration to advance health equity by improving the experiences of SGM people living with dementia and their care partners?

### Step 2: Identifying Relevant Studies

Relevant studies will be identified through a comprehensive database search conducted by health librarians. Two reviewers will independently screen titles and abstracts and review full-text articles to identify relevant studies using predefined eligibility criteria.

#### Eligibility Criteria

Eligible studies will include participants living with dementia who identify as SGM, SGM people living with dementia *and* their care partners, or SGM care partners caring for people living with dementia. Dementia will be broadly defined as any self-report or medical diagnosis of dementia of any type or severity. Studies focused on mild cognitive impairment will not be included. Studies will focus on the phenomenon of interest, which is the health and health care experiences of participants. The studies must be in English; the 2 researchers conducting the review are English speakers. Studies will not be excluded based on research methods (eg, qualitative, quantitative, and mixed), setting (eg, hospital, long-term care, and community), or publication date. Dissertations, conference proceedings, working papers, and non-English studies will be excluded.

#### Search Strategy

The following databases will be searched to identify relevant studies: MEDLINE (PubMed), Embase (Elsevier), CINAHL (EBSCOhost), AgeLine (EBSCOhost), PsychINFO (EBSCOhost), and Scopus (Elsevier). These databases were selected by the authors with guidance of a health librarian to ensure that a wide variety of publications from various fields are located. A combination of keywords and database-specific subject headings for the concepts of gender or sexuality and dementia will be used. No restrictions will be placed by date in the database search. A health librarian will assist in developing the keyword search and conduct the search strategy. A draft of what the search might look like for PubMed with relevant combinations of Medical Subject Headings and keywords is included in Table 1. Additionally, a health librarian will conduct a separate search of the grey literature to identify relevant policy papers, white papers, blog posts, and op-eds [23]. Electronic database searches will occur within the same week and extracted into Covidence (Veritas Health Innovation) software [24]. Covidence will be used to organize, review, and remove duplicate citations.

**Table 1:** Draft PubMed search strategy.

(1) Gender or sexuality key terms	“Bisexuality”[Mesh^a^] OR “Homosexuality, Male”[Mesh] OR “Homosexuality”[Mesh] OR “Homosexuality, Female”[Mesh] OR “Sexual behavior”[Mesh] OR “Sexual and Gender Minorities”[MeSH Terms] OR “Transgender Persons”[Mesh] OR “Health Services for Transgender Persons”[Mesh] OR ‘Intersex Persons”[Mesh] OR “Transsexualism”[Mesh] OR MSM[tiab] OR BMSM[tiab] OR “men having sex with men”[tiab] OR “men who have sex with men”[tiab] OR “men who have sex with other men”[tiab] OR “women having sex with women”[tiab] OR “women who have sex with women”[tiab] OR “women who have sex with other women”[tiab] OR bisexual[tiab] OR bisexuals[tiab] OR bisexuality[tiab] OR homosexual[tiab] OR homosexuals[tiab] OR homosexuality[tiab] OR “homo sexual”[tiab] OR “homo sexuals”[tiab] OR gay[tiab] OR gays[tiab] OR lesbian[tiab] OR lesbians[tiab] OR lesbianism[tiab] OR sexuality[tiab] OR Trans[tiab] OR transgender[tiab] OR transgenders[tiab] OR transgenderism[tiab] OR transgendered[tiab] OR transsexual[tiab] OR transsexualism[tiab] OR transsexuals[tiab] OR transsexuality[tiab] OR transvestite[tiab] OR transvestites[tiab] OR transvestism[tiab] OR transman[tiab] OR transmen[tiab] OR transwoman[tiab] OR transwomen[tiab] OR Intersex[tiab] OR intersexuality[tiab] OR intersexual[tiab] OR intersexuals[tiab] OR “Inter sex”[tiab] OR “inter sexuality”[tiab] OR “inter sexual”[tiab] OR “inter sexuals”[tiab] OR “two spirit”[tiab] OR “two-spirit”[tiab] OR “two nature”[tiab] OR “two-nature”[tiab] OR hijra[tiab] OR “third gender”[tiab] OR “third sex”[tiab] OR nonbinary[tiab] OR non-binary[tiab] OR “non binary”[tiab] OR “same sex”[tiab] OR “same-sex”[tiab] OR asexual[tiab] OR asexuals[tiab] OR asexuality[tiab] OR Pansexual[tiab] OR pansexuals[tiab] OR pansexuality[tiab] OR polyamory[tiab] OR polyamorous[tiab] OR polysexual[tiab] OR polysexuals[tiab] OR polysexuality[tiab] OR queer[tiab] OR queers[tiab] OR genderqueer[tiab] OR nonheterosexual[TIAB] OR “non-heterosexual”[TIAB] OR “non heterosexuals”[TIAB] OR nonheterosexuals[TIAB] OR LGBT[tiab] OR LGBTQ[tiab] OR LGBTQIA[tiab] OR LGBTQIA+[tiab] OR GLBT[tiab] OR TGNB[tiab] OR ((transition[tiab] OR transitioning[tiab] OR transitioned[tiab] OR transitions[tiab]) AND (female[tiab] OR male[tiab] OR woman[tiab] OR women[tiab] OR men[tiab] OR man[tiab] OR females[tiab] OR males[tiab])) OR ((Gender[tiab] OR sexual[tiab]) AND (behavior[tiab] OR behaviors[tiab] OR behaviour[tiab] OR behaviours[tiab] OR “non conforming”[tiab] OR “non-conforming”[tiab] OR fluid[tiab] OR orientation[tiab] OR presentation[tiab] OR identity[tiab] OR identities[tiab] OR minority[tiab] OR minorities[tiab] OR diverse[tiab] OR diversity[tiab] OR dysphoria[tiab] OR dysphoric[tiab] OR dysphorias[tiab] OR disorder[tiab] OR disorders[tiab] OR change[tiab] OR affirmation[tiab] OR confirmation[tiab] OR reassignment[tiab] OR reassigned[tiab] OR change[tiab] OR changed[tiab] OR changes[tiab] OR changing[tiab] OR expansive))
(2) Dementia key terms	“AIDS Dementia Complex”[Mesh] OR “Alzheimer Disease”[Mesh] OR “Aphasia, Primary Progressive”[Mesh] OR “Primary Progressive Nonfluent Aphasia”[Mesh] OR “Creutzfeldt-Jakob Syndrome”[Mesh] OR “Dementia, Vascular”[Mesh] OR “Dementia, Multi-Infarct”[Mesh] OR “Diffuse Neurofibrillary Tangles with Calcification”[Mesh] OR “Frontotemporal Lobar Degeneration”[Mesh] OR “Frontotemporal Dementia”[Mesh] OR “Primary Progressive Nonfluent Aphasia”[Mesh] OR “Huntington Disease”[Mesh] OR “Kluver-Bucy Syndrome”[Mesh] OR “Lewy Body Disease”[Mesh] OR dementia[tiab] OR dementias[tiab] OR Alzheimer[tiab] OR alzheimers[tiab] OR alzheimer's[tiab] OR aphasia[tiab] OR aphasias[tiab] OR creutzfeldt-jakob[tiab] OR “creutzfeldt Jakob”[tiab] OR CADASIL[tiab] OR “diffuse neurofibrillary tangles”[tiab] OR “frontotemporal lobar degeneration”[tiab] OR “frontotemporal lobar degenerations”[tiab] OR “huntington disease”[tiab] OR huntingtons[tiab] OR huntington’s[tiab] OR kluver-bucy[tiab] OR “kluver bucy”[tiab] OR “lewy body”[tiab] OR amentia[tiab] OR amentias[tiab]
(3)	1 AND 2
(4)	3 NOT (Editorial[pt] OR Letter[pt] OR Comment[pt])

^a^MeSH: Medical Subject Headings.

### Step 3: Study Selection

Titles and abstracts will be reviewed independently by 2 reviewers (JTM and MLH) according to the defined eligibility criteria. Weekly meetings will be held to resolve discrepancies and reach consensus on final titles and abstracts. Each author will then independently review all full-text articles to determine eligibility. Discrepancies will be resolved through discussion between the 2 reviewers.

### Step 4: Charting the Data

**Figure 1:**
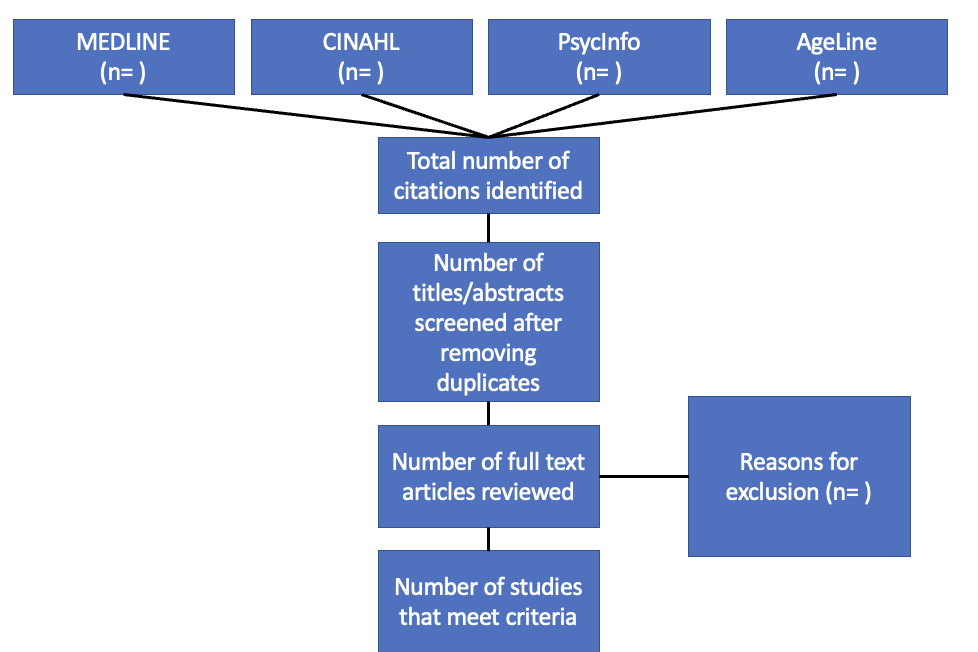
PRISMA flow diagram of the studies to be included in the scoping review.

Covidence will record the number of articles included, excluded, and the reasons for exclusion, which will be used to develop a PRISMA-ScR flowchart (Figure 1).

#### Data Extraction

A data extraction tool will be used to collect publication details, and information pertaining to study characteristics, findings, and author conclusions of included studies. Textbox 1 shows information to be included in the data extraction tool. The tool will be modified as needed during the data extraction phase; these changes will be reported in the final scoping review.

**Information gathered using data extraction tool.**

**Publication details**

- Author
- Year of publication
- Country of origin
- Publication type

**Study characteristics**

- Aims or purpose
- Study design
- Methods used
- Participants
- Sample size
- Setting
- Recruitment strategy

**Key findings**

- Qualitative findings
- Quantitative results
- Context to which the findings apply (health or health care experiences? Community vs health care system?)

**Author conclusions**

- Study limitations
- Implications
- Recommendations for research
- Recommendations for policy
- Recommendations for practice

#### Data Management

The 2 reviewers will independently extract data manually from each study and chart it in Microsoft Excel spreadsheets. Data extraction tables will be compared through regularly scheduled team meetings.

### Step 5: Collating, Summarizing, and Reporting Results

#### Data Analysis Procedure

Following data extraction, we will use the data extraction tables to identify key information that will be collated in a comprehensive summary table. The summary table will include author and year, study aims or purpose, study design and methods, country, sample, key findings, limitations, implications, and recommendations. In the summary table, a brief description will be added regarding the sociopolitical climate surrounding SGM populations for each study’s country of origin. This information will be gathered from the article itself and by analyzing survey data reports from reputable sources like Pew Research Center and The Williams Institute at University of California, Los Angeles School of Law. Content and structure of the summary table may change based on the results of the data extraction process.

#### Data Analysis

To further synthesize results of the included studies, each reviewer will independently analyze each study and the tabulated summary data to identify common concepts and themes across studies. Concepts and themes will be discussed through regular team meetings, and discrepancies will be resolved through discussion. Reviewers will finalize and define each concept and theme together. A table of themes, concepts, definitions, and exemplars (eg, qualitative findings, quantitative results, and key statements) from the included studies to support each theme will be developed (Table 2 is an example of a themes table).

**Table 2:** Example of a table depicting themes, concepts, definitions, and exemplars identified through thematic analysis.

Major theme name 1 (theme definition, as defined by thematic analysts MLH and JTM)			
Concepts	Concept definition	Support or exemplars	Paper
Concepts identified through thematic analysis	Defined by thematic analysts (MLH and JTM)	Raw quantitative data or results Qualitative quotes and themes or subthemes Key statements from author conclusions	Author and year

### Step 6: Consultation

The final step in the Joanna Briggs Institute protocol for scoping reviews is consultation. With support and guidance from the authors’ institutional Community-Engaged Research Initiative, a stakeholder group of SGM care partners and people living with dementia will be developed, and a listening session will be convened. Stakeholders will be recruited from, for example, SGM-friendly church organizations, SGM and dementia care support groups, and SGM or dementia community organizations. Stakeholder participants will be compensated for their time with a gift card. The listening session will include a presentation of the scoping review themes, followed by open discussion for stakeholders to provide feedback on the findings. Stakeholders will be asked to discuss whether the themes resonate with their personal experiences, and they will be asked to identify topics that were left out of the scoping review themes. They will then be asked to propose key recommendations for future research, policy, and practice initiatives based on their own lived experiences and the scoping review themes. Stakeholder feedback provides those who are affected by these experiences with relevant research and findings [25].

Detailed notes will be kept by 2 trained notetakers during the listening session. MLH and JTM will compare and contrast the topics discussed or the notes taken during the listening session with the original themes identified through thematic analysis. A series of meetings will be held to refine and synthesize themes, gaps, and recommendations based on the combined results of the thematic analysis and listening session. The themes table (Table 2) will be updated based on results of the synthesis meetings. Findings generated through this consultation process are anticipated to inform future research, policy, and practice initiatives focused on SGM people living with dementia and their care partners.

## Results

Results of this review will include a PRISMA-ScR flow diagram to illustrate results of the database search and the final included studies. Key study information for all included studies will be presented in narrative and tabular format. Integrated findings from the thematic analysis and listening session will be presented through a narrative synthesis with detailed description of the final themes, gaps, and recommendations. Finalized themes, definitions, and exemplars from the thematic analysis and listening session will also be depicted in a table. We will present a discussion regarding the significance of the findings of this scoping review as well as concrete recommendations to inform future research, policy, and practice to improve health and health care experiences of SGM people living with dementia and their care partners.

## Discussion

### Overview and Expected Outcomes

Older adults living with dementia and their care partners who identify as SGM are at the intersection of several identities associated with disparities in health outcomes and health care provision [2-4,26]. Understanding the health and health care experiences of individuals and families living at the intersection of such identities is critical to inform practice and policy initiatives to address health disparities and advance health equity. No prior reviews have focused on synthesizing the evidence relating to the health and health care experiences of SGM people living with dementia and their care partners. This review will characterize the extent and nature of the research surrounding SGM people living with dementia and their care partners and identify meaningful gaps that warrant future examination. Findings of this review are anticipated to act as a stepping stone to inform a future program of action-oriented research aimed to address health inequities experienced by SGM people living with dementia and their care partners.

The innovation of this review is the use of community-engaged methods to address the consultation step of the scoping review framework, which is currently considered an optional component [27]. Engaging members of a target population early in the research process is pivotal to uncovering the experiences and needs of groups that have historically been socially and economically marginalized. Given the focus of this review on members at the intersection of multiple stigmatized identities, convening a listening session with stakeholders who identify as SGM and are living with or caring for someone with dementia will be critical to not only identify disparities but also elucidate potential paths to advance health equity. Integrated findings from the scoping review and the community-engaged listening session are likely to yield concrete recommendations and areas to explore to address health inequities experienced by this population. Other strengths of this review include collaboration with health librarians in building and conducting the database search, searching multiple databases across several disciplines (medicine, nursing, and gerontology), and including 2 independent reviewers in the screening process.

Potential limitations of this review include the lack of critical appraisal of included studies, which would provide deeper insight into the overall quality of research. Additionally, this review will not contribute essential data to advance equity; it will instead focus on synthesizing the extent and nature as well as gaps in this area of research. Finally, although a comprehensive database search will be conducted, it is still possible that studies will be missed, as the grey literature and non-English studies will be excluded. These limitations notwithstanding, findings of this review will be the first to summarize the research focused on SGM people living with dementia and their care partners, and they are likely to yield concrete areas to explore through future efforts in research, policy, and practice.

### Future Directions

Findings from this review will be used to inform a future program of research focused on advancing equity for SGM families living with dementia. Based on what is learned in relation to the state of the science, further exploration through qualitative inquiry to understand disparities may be warranted, while recommendations from key stakeholders will be used to directly inform action-oriented pilot studies aimed at addressing gaps in health and health care of this population. Community-engaged methods used in this review will provide a foundation to inform the design of future studies as well as policy initiatives focused on advancing health equity for aging adults.

### Conclusions

In this scoping review, we aim to understand the health and health care experiences of SGM people living with dementia and their care partners. This study may provide a deeper understanding of the health disparities and challenges navigating the health care system for this population. We hope to gain insight for future studies and the development of interventions that advance health equity for SGM people living with dementia and their care partners.

## Abbreviations

PRISMA-ScR: Preferred Reporting Items for Systematic Reviews and Meta-Analyses extension for Scoping Reviews
SGM: sexual and gender minorities

## Data Availability

Data sharing is not applicable to this paper, as no data sets were generated or analyzed during the current study.
